# Does Optic Nerve Head Surface Topography Change Prior to Loss of Retinal Nerve Fiber Layer Thickness: A Test of the Site of Injury Hypothesis in Experimental Glaucoma

**DOI:** 10.1371/journal.pone.0077831

**Published:** 2013-10-25

**Authors:** Brad Fortune, Juan Reynaud, Lin Wang, Claude F. Burgoyne

**Affiliations:** Discoveries in Sight Research Laboratories, Devers Eye Institute, and Legacy Research Institute, Legacy Health, Portland, Oregon, United States of America; University of Houston, United States of America

## Abstract

**Purpose:**

To test the hypothesis that optic nerve head (ONH) deformation manifesting as changes in its mean surface height precedes thinning of the peripapillary retinal nerve fiber layer (RNFL) in experimental glaucoma (EG).

**Methods:**

68 rhesus macaque monkeys each had three or more baseline imaging sessions under manometric intraocular pressure (IOP) control to obtain average RNFL thickness (RNFLT) and the ONH surface topography parameter mean position of the disc (MPD). Laser photocoagulation was then applied to the trabecular meshwork of one eye to induce chronic, mild-to-moderate IOP elevation and bi-weekly imaging continued. Event analysis was applied to determine for each parameter when an ‘endpoint’ occurred (signficant change from baseline) for eight different endpoint criteria. Specificity was assessed in the group of 68 fellow control eyes. Classical signal detection theory and survival analysis were used to compare MPD with RNFLT.

**Results:**

Regardless of the endpoint criterion, endpoints were always more frequent for MPD than for RNFLT. The discriminability index (d’) was 2.7 ± 0.2 for MPD and 1.9 ± 0.2 for RNFLT (p<0.0001). Endpoints were reached by MPD an average of 1-2 months earlier than by RNFLT (p<0.01). At the onset of the first specific, detectable MPD change in EG eyes, there was still no significant change in RNFLT on average (p=0.29) and only 25% of individual eyes exhibited signficant reduction. In contrast, at onset of signficant RNFLT change, MPD had already changed an average of 101 µm from baseline (p<0.0001) and 71% of the individual eyes had exhibited significant change. The magnitude of MPD change was more than could be explained on the basis of axon loss alone.

**Conclusions:**

This study demonstrates that the average surface height of the ONH changes prior to any detectable loss of average peripapillary RNFL thickness in non-human primate eyes with experimental glaucoma.

## Introduction

Vision loss in glaucoma is thought to occur primarily because retinal ganglion cell axons are injured within the optic nerve head (ONH) [1-4] by one or more of several possible mechanisms including disruption of axonal transport [1,5-8], dysregulation of optic nerve head blood flow [9,10], mitochondial dysfunction [11], alterations of glial cell homeostasis [12-15] and perhaps also other, unidentified, direct mechanical effects on the axons themselves such as stretch [16,17]. Though chronic progressive deformation of the ONH (traditionally referred to as “cupping”) is commonly considered the hallmark of glaucoma, a clinical sign used to distinguish it from other optic neuropathies [18-22], the extent of deformation can vary from eye to eye relative to the stage of damage. For example, the “senile sclerotic” form of glaucomatous cupping [23,24] is thought to represent relatively less ONH deformation for a given stage of axon and vision loss [25]. This suggests that the process of axonal injury can still occur in the presence of relatively less ONH deformation on a macro scale (while on a smaller scale, deformation of individual laminar beams and the lamina “pores” through which the axon bundle traverse might still be present despite lesser clinical evidence of “cupping”) [25]. The dissociation between the degree of ONH deformation and the extent of axon or vision loss in part has driven investigations into alternative theories about the site of retinal ganglion cell injury in glaucoma. 

Results of previous collaborative studies carried out in our laboratories suggest that ONH deformation manifest as ONH surface topography changes precedes the loss of axons that can be detected by thinning of the peripapillary RNFL in a non-human primate model of experimental glaucoma. For example, Yang et al reported that there was an average of only ~20% loss of axons from the orbital optic nerve 3-6 weeks after the onset of ONH surface topography change in monkeys with experimental glaucoma [26]. Strouthidis et al found that RNFL thickness had not changed from baseline (or versus fellow control eyes) at a slightly earlier stage (at the onset of ONH surface topography change) [27]. Fortune et al specifically analyzed this time point in a larger pool of subjects and reported similarly that there was no change in RNFL thickness, on average, at onset of surface topography change [28]. These findings seem to suggest that ONH deformation precedes axon loss in non-human primate experimental glaucoma because if axon loss were the only contributor to ONH deformation (i.e. the null hypothesis, in the absence of remodelling and/or changes in connective tissue architecture, glial cell morphology and/or proliferation, loss of capillaries, etc.), then thinning of the peripapillary RNFL should occur in concert (for both time and magnitude) with retreat of the ONH surface. Similarly, this hypotheis would predict that optic disc changes precede vision loss when both are followed longitudinally [29,30].

However, in our prior studies, the definition of ONH surface topography change was allowed to be focal (versus global) and included subjective determinations (albeit by masked analysis), whereas peripapillary RNFL thickness changes were based on global, quantitative measurements. We wished to make this comparison more formally, from a position of greater equipose. Therefore in the present study, we compare longitudinal quantitative measurements of ONH surface topography and peripapillary RNFL thickness to test the hypothesis that ONH deformation – manifesting as ONH surface height changes – precedes thinning of the peripapillary RNFL in experimental glaucoma. 

## Materials and Methods

### Subjects

The subjects of this study were 68 rhesus macaque monkeys (*Macaca mulatta*), 56 female and 12 male. Their average age (± standard deviation, SD) was 10.2 ± 5.8 years, ranging 1.2 to 22.6 years. This study was carried out in strict accordance with the recommendations in the Guide for the Care and Use of Laboratory Animals of the National Institutes of Health and were approved and monitored by the Institutional Animal Care and Use Committee (IACUC) at Legacy Health (USDA license 92-R-0002 and OLAW assurance A3234-01). All efforts were made to minimize suffering. Animals are pair-housed unless socially incompatible or an IACUC approved, scientifically justified exemption exists. Nutritionally complete Purina Lab Diet monkey biscuits are fed twice daily. In addition, animals are provided with various fruits and vegetables as supplements. The IACUC approved environmental enrichment plan includes regular access to enrichment cages, various manipulanda (Kong toys, hard wood, mirrors, etc.), foraging devices, daily interaction with animal care staff and access to scheduled audio and/or video within the animal room. 

### Anesthesia

All experimental procedures began with induction of general anesthesia using ketamine (15 mg/kg IM) in combination with either xylazine (0.8-1.5 mg/kg IM) or midazolam (0.2 mg/kg IM), along with a single subcutaneous injection of atropine sulphate (0.05 mg/kg). Animals were then intubated and breathed isoflurane gas (1-2%; typically 1.25%) mixed with oxygen to maintain anesthesia during all imaging procedures. A clear, rigid gas permeable contact lens filled with 0.5% carboxymethylcellulose solution was placed over the apex of each cornea. Heart rate and arterial oxyhemoglobin saturation were monitored continuously and maintained above 75 per min and 95%, respectively. Body temperature was maintained at 37 ° C. 

### Optic Nerve Head Surface Topography – Mean Position of the Disc (MPD)

ONH surface topography was measured by confocal scanning laser tomography (CSLT) using a Heidelberg Retina Tomograph II (Heidelberg Engineering GmbH, Heidelberg, Germany) as recently described [27,28]. A minimum of three individual scans were acquired at each CSLT imaging session and averaged to create a mean topography for each eye. All CSLT scans were performed 30 minutes after IOP was manometrically lowered to 10 mmHg because the ambient IOP level is known to significantly influence ONH structure and surface topography [31-34]. Thus, elastic (reversible) deformation of ONH tissues due to IOP elevation existing at the start of any given imaging session was eliminated, leaving primarily only the permanent changes to influence the topography [31,35].

A trained technician outlined the optic disc margin within the baseline image of each eye using a disc photograph for reference where necessary; this contour line was automatically transferred to all subsequent images in the longitudinal series. For the current study, the parameter mean position of the disc (MPD) was calculated for each CSLT session as described previously [31]. Briefly, MPD refers to the height of the surface of the ONH (i.e. average height of all pixels located within the disc margin contour line) relative to the height of a standard reference plane. The MPD value used in this analysis was derived from the averaged topography. The CSLT quality score (standard deviation of pixel height in mean image) was ≤20 for 97.7% of the 3490 scans used in this study, ≤30 for 99.3% of scans and >40 for 0.3% of scans (median score was 10). 

### Peripapillary RNFL Thickness

Peripapillary RNFL thickness was measured using spectral domain OCT (SDOCT, Spectralis, Heidelberg Engineering GmbH) as previously described [28]. For this study, the average peripapillary RNFL thickness was measured from a single circular B-scan consisting of 1536 A-scans. Nine to sixteen individual sweeps were averaged in real time to comprise the final stored B-scan at each session. The position of the scan was centered on the ONH at the first imaging session and all follow-up scans were acquired in this same location using the instrument’s automatic active eye tracking software. A trained technician masked to the purpose of this study manually corrected the accuracy of the instrument’s native automated layer segmentations when the algorithm had obviously erred from the inner and outer borders of the RNFL to an adjacent layer (such as a refractive element in the vitreous instead of the internal limiting membrane, or to the outer border of the inner plexiform layer instead of the RNFL). The SDOCT quality score was ≥20 for 97.7% of the 3436 scans used in this study, ≥30 for 55.6% of scans and <15 for 0.1% of scans (median score was 31). 

### Intraocular Pressure (IOP) Measurements

IOP was measured in both eyes at the start of every session using a Tonopen XL (Reichert Technologies, Inc., Depew, NY). The value recorded for each eye was the average of three successive measurements. 

### Experimental Design and Protocol

Each animal had a minimum of three weekly baseline recordings for each of the above-described imaging sessions; the average number of pre-laser baseline sessions (± SD) was 5.8 ± 2.6. Argon laser photocoagulation was then applied to the trabecular meshwork of one eye of each animal to induce chronic elevation of IOP [36,37]. Initially, 180 deg of the trabecular meshwork was treated in one session, then the remaining 180 deg was treated in a second session approximately two weeks later. If necessary, laser treatments were repeated in subsequent weeks (limited to a 90° sector) until an IOP elevation was first noted or if the intitial post-laser IOP had returned to normal levels. The average number of laser treatments (± standard deviation) was 5.4 ± 3.2. 

HRT and SDOCT imaging was repeated every other week during the post-laser follow-up period, always under manometric IOP control (i.e. 30 minutes after setting IOP to 10 mmHg in both eyes). Imaging continued for each animal until its pre-defined sacrifice target had been reached. Specific targets for the EG stage when each animal was sacrificed were based on the primary study to which each animal was assigned and were pre-determined based on those protocols. Thus the EG stage at sacrifice and details of sacrifice procedures differed across animals. In all cases death occurred by exsanguination under deep anesthesia (overdose of pentobarbital in 33 of the 68 animals and under deep isoflurane anesthesia in 35 of 68). 

### Analysis and Statistics

An ‘event analysis’ was used to compare the time (from first laser) to reach an endpoint for ONH surface topography change measured as MPD versus the time to reach an endpoint for RNFL thickness. Two alternative approaches were compared for this analysis. The first approach was to calculate the 95% confidence limits of baseline variability for each individual eye using the Student’s t-distribution with *n*-1 degrees of freedom, where *n* represents the number of pre-laser baseline observations for each animal. The endpoint event was defined by four alternative criteria as follows: 1) the first observation below the lower limit of baseline variability (thus no confirmation required); 2) the second of two consecutive observations below the lower limit of baseline variability (thus based on a single sequential confirmation); 3) the third of three consecutive observations below the lower limit of baseline variability (thus based on two sequential confirmations); 4) the third observation below the lower limit of baseline variability within any sequence of four time points (thus based on two confirmations, but not necessarily sequential). 

The second approach was to estimate measurement noise for each parameter based on all available data from all pre-laser baseline observations of all eyes in the study and to use that measurement noise estimate to define the lower limit of baseline variability for each individual eye. In this analysis, the 95% limits of each parameter’s measurement noise was determined by bootstrapping from all possible pairwise differences of pre-laser baseline observations within each of the 136 study eyes. In each iteration of the bootstrap, one baseline pairwise difference was sampled from each of the 136 eyes and the empirical 95% confidence limits were recorded as a percentage of the mean parameter value; the final limit was taken as the average of 1000 such iterations for each RNFL parameter. Thus, the lower limit of variability from pre-laser baseline values (i.e. the threshold for significant change) was defined for each eye as its pre-laser baseline average minus the 95% confidence limit of measurement noise for that parameter. The same four criteria listed above were applied to define the endpoint event in this second approach. Specificities were assessed in the group of 68 fellow (non-lasered) control eyes for both approaches and each endpoint definition. Classical signal detection theory [38] was applied to compare the parameter MPD against the parameter RNFL thickness using all eight endpoint criteria described above (four criteria within each of the two approaches). All statistical analysis was performed using a commercial software package (Prism 5, GraphPad Software, Inc., La Jolla, CA). 

## Results

The average duration of post-laser follow-up (± SD) was 10.0 ± 7.4 months (range: 2.0 to 36.9 months). Mean IOP over the span of post-laser follow-up ranged from 9.6 to 35.5 mmHg in the group of 68 EG eyes, with a group average of 19.8 ± 6.0 mmHg. Mean IOP in the group of 68 fellow control eyes was 11.4 ± 1.9 mmHg. The peak IOP observed during post-laser follow-up period was 40.6 ± 11.6 in the EG eyes (range: 15.3 to 61.0 mmHg) and 17.4 ± 3.4 mmHg in control eyes. The magnitude of RNFL thickness loss at the final available time point (relative to baseline) was associated with both mean IOP (R^2^ = 0.32, p<0.0001, N=68 EG eyes) and peak IOP (R^2^ = 0.26, p<0.0001). The degree of posterior deformation of the ONH surface measured by MPD at the final available time point (relative to baseline) was also associated with both mean IOP (R^2^ = 0.34, p<0.0001) and peak IOP (R^2^ = 0.21, p<0.0001). 

**Figure 1:**
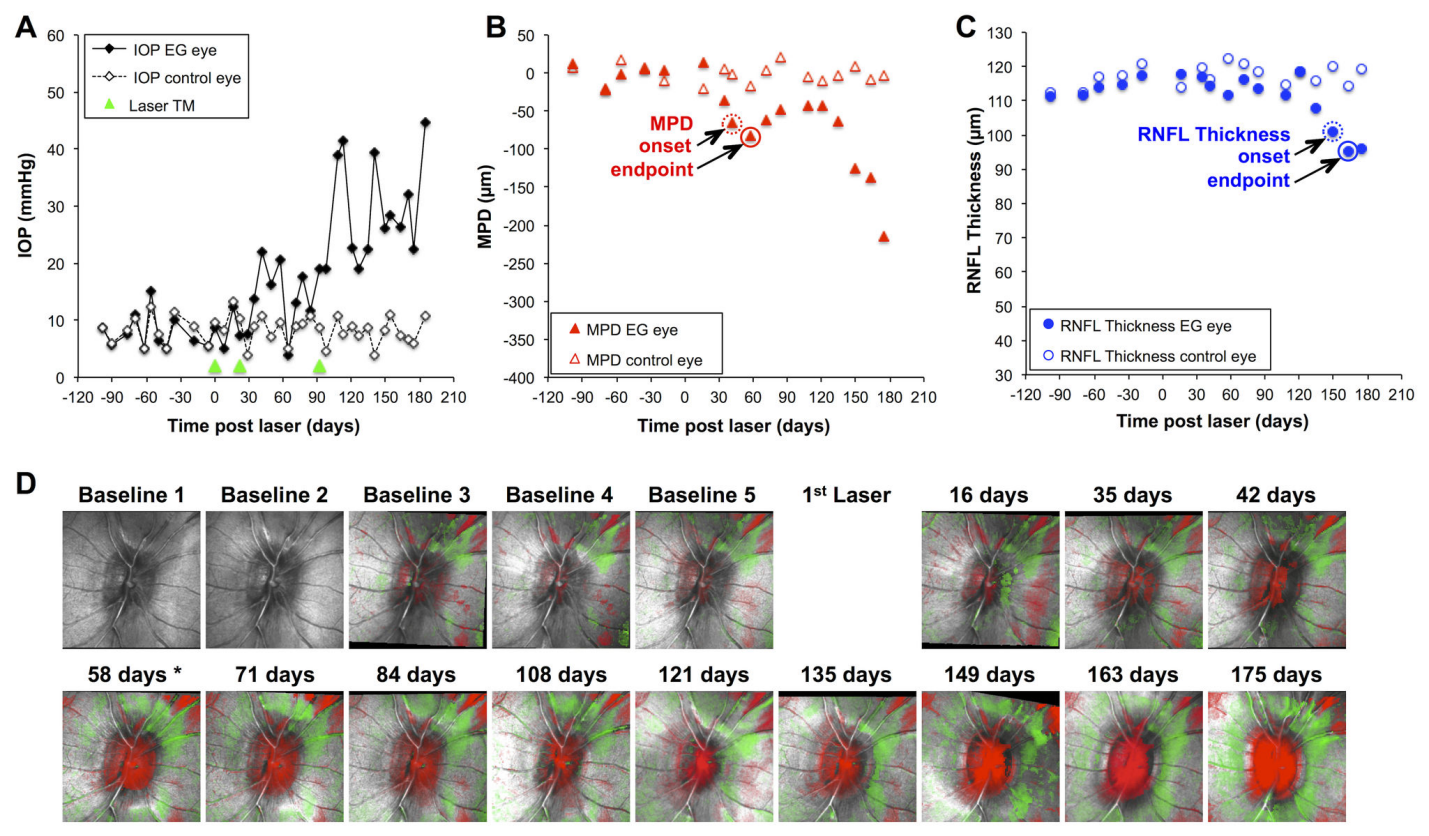
Longitudinal comparison of optic nerve head surface topography and peripapillary retinal nerve fiber layer (RNFL) thickness in a single representative animal. (**A**) IOP versus time; green arrowheads indicate dates of laser photocoagulation to the trabecular meshwork (Laser TM) in the eye with experimental glaucoma (EG, filled diamonds) and fellow control eye (open diamonds). (**B**) Optic nerve head surface topography parameter “mean position of the disc” (MPD) versus time in the EG eye (filled triangles) and fellow control eye (open triangles). (**C**) Peripapillary RNFL thickness versus time in the EG eye (filled circles) and fellow control eye (open circles). The data points indicated by closed circles in 1B and 1C represent the endpoint for MPD and RNFL thickness, respectively, determined by the criterion of two sequential observations below the measurement noise limit; the data points indicated by broken circles represent the “onset” determined by the same criterion. (**D**) Topographic Change Analysis (TCA) maps are shown for the EG eye; red superpixels represent significant posterior deformation as compared with the first baseline, green superpixels represent significant anterior deformation. Asterisk indicates date of MPD endpoint shown in 1B, 58 days after first laser.

Figure 1 shows the longitudinal data for one representative individual example. IOP was consistently elevated in the EG eye of this animal after three laser applications to the trabecular meshwork (Figure 1A). Over the duration of post-laser follow-up, IOP averaged 20 mmHg in the EG eye, with a peak of 41 mmHg. The parameter MPD is plotted versus time in Figure 1B and RNFL thickness versus time in Figure 1C. For reference, the Topographic Change Analysis (TCA) [39] maps are shown for the EG eye in Figure 1D, where red superpixels represent significant posterior deformation of the local ONH surface height relative to baseline. The circled data point in Figures 1B and 1C represents the endpoint for MPD and RNFL thickness, respectively, determined by the twice sequential criterion (i.e. single confirmation) beyond the measurement noise estimate of baseline variability limits (i.e. variability limits based on all eyes). These measurement noise limits (95%) were calculated to be ±52 µm from baseline average for MPD and ±7.0% from baseline average for RNFL thickness, both of which are very close to previous calculations based on smaller samples [28,40]. Thus, for this twice-sequential criterion, the endpoint for MPD occurred at 58 days post-laser and the endpoint for RNFL thickness occurred at 163 days post-laser in this EG eye. The fellow control eye of this animal did not meet any of the eight criteria for endpoint. 

**Figure 2:**
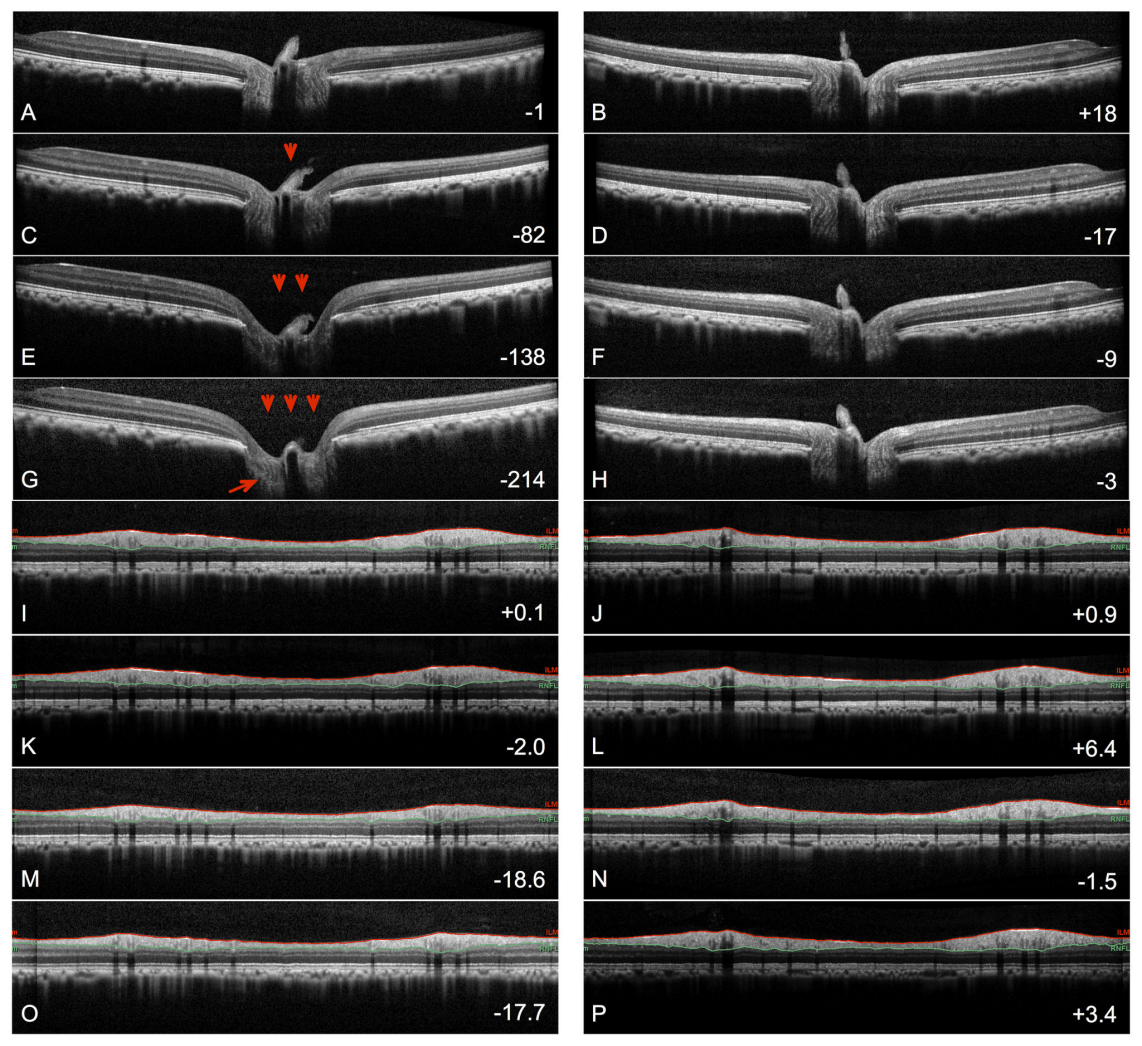
Longitudinal series of SDOCT B-scans crossing through the optic nerve head and fovea in the EG eye (A,C,E,G) and fellow control eye (B,D,F,H) of the same animal shown in Figure 1. The four time points shown are: baseline 3 (A,B), MPD endpoint (C,D), RNFL thickness endpoint (E,F) and the final available time point (G,H). (**I**-**P**) SDOCT peripapillary circular B-scans obtained at the same set of four time points. The numbers in the lower right corner of each panel represent the change from baseline average (in µm) for MPD (panels A-H) and RNFL thickness (panels I-P). Signficant posterior deformation of the ONH surface occurred in the EG eye (red arrowheads) prior to significant change in RNFL thickness. Posterior deformation of the lamina cribrosa was evident in the scans through the ONH (red arrow).

The differences between longitudinal changes in ONH surface height and peripapillary RNFL thickness can also be appreciated by evaluating cross-sectional views of the ONH using SDOCT line scans. Figure 2 shows one B-scan (from a total scan pattern consisting of 80 radial B-scans centered on the ONH) at one of the baseline time points (baseline 3) in both eyes of this animal in the top row. Repeat scans at the same location are shown for subsequent time points in both the EG eye (left column) and fellow control eye (right column). The second row shows the scans obtained on the date determined to be the MPD endpoint for the twice-sequential criterion and whole cohort estimate of measurement noise (58 days post laser; see Figure 1B). The third row shows scans obtained on the date determined to be the RNFL thickness endpoint by the same criterion (163 days post laser; see Figure 1C) and the fourth row shows the scans from the final available time point (175 days post laser). The peripapillary circular B-scans obtained at the same time points are shown in the panels I - P. The numbers in the lower right corner of each panel represent the change from baseline average (in µm) for MPD (panels A-H) and RNFL Thickness (panels I-P). Figure 2 shows that in this eye, there was only a 2 µm (1.7%) decrease in RNFL thickness at the MPD endpoint determined by this criterion (Figure 2C and 2K). In contrast, by the time an RNFL thickness endpoint was reached by the same criterion (Figure 2M), the ONH surface has already undergone substantial posterior deformation, such that the MPD parameter was 138 µm posterior to the baseline average. At the final time point, there was an even larger disrepancy between MPD and RNFL thickness since the ONH surface had moved even farther posterior (MPD was -214 µm) without any signficant further change for RNFL thickness. 

**Figure 3:**
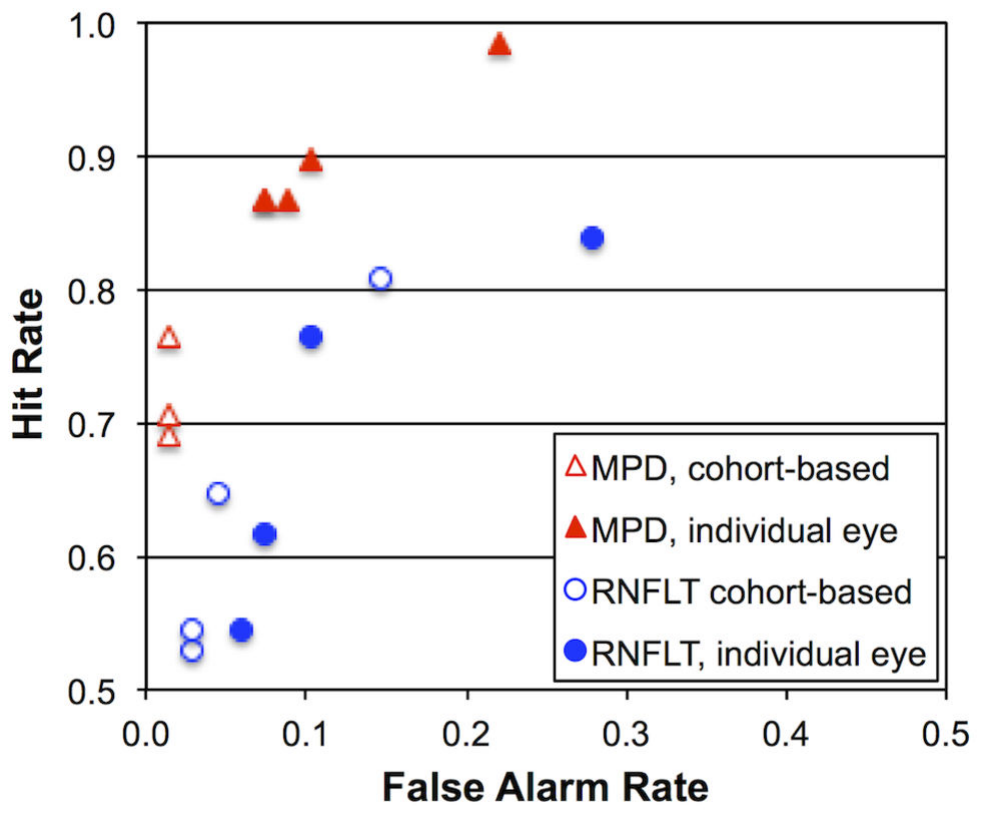
Receiver-operating characteristic (ROC) plot comparing the parameter MPD versus the average peripapillary RNFL thickness for the eight different endpoint criteria. Hit rate, which represents the proportion of EG eyes reaching endpoint by a given criterion, is plotted against false alarm rate, which represents the proportion of fellow control eyes reaching endpoint by the same criterion. Note, this plot is truncated at 0.5 along both axes, so it represents only the top left quadrant of a complete ROC plot.

Classical signal detection theory [38] was applied in order to compare MPD and RNFL thickness endpoints simultaneously for all endpoint criteria. Thus, for each endpoint criterion, the proportion of EG eyes meeting the endpoint definition was calculated to represent a hit rate and the proportion of fellow control eyes meeting the same endpoint definition was calculated to represent the false alarm rate. Figure 3 plots hit rate versus false alarm rate for each of the eight endpoint definitions. It demonstrates that the hit rate for MPD at any given false alarm rate was always higher than the hit rate for RNFL thickness. This means that regardless of the definition chosen for endpoint criterion, there were always more endpoints observed for MPD than for RNFL thickness. The discriminability index (d’) was calculated for each of the eight endpoint definitions and found to be 2.7 ± 0.2 for MPD and 1.9 ± 0.2 for RNFL thickness (p<0.0001). 

The data in Figure 3 also demonstrate that the individual eye approach to determining baseline variability limits results in a higher false-alarm rate for any given criterion than does the approach based on estimating measurement noise from the entire cohort’s data. This latter result is consistent with the central limit theorum and the greater likelihood that the baseline series for any given individual eye will contain samples closer to the mean than it will extremes. Thus the limits estimated from individual series are more likely to be too “tight” than too “liberal”, that is, the individual eye series are more likely to provide an underestimate than they are an overestimate of true baseline variability. For the remainder of this study, the criterion chosen to define endpoint for all other analyses was as shown in Figure 1, namely, two-sequential observations below the lower limit of baseline variability based on the measurement noise estimate calculated from the entire cohort’s data. For both MPD and RNFL thickness, this criterion was close to the point perpendicular to the no discrimination locus (d’=0), representing a good balance between criterion effects; the largest hit rate without a notable increase in false alarm rate. 

Using this criterion, 52 (76.5%) of EG eyes reached endpoint for MPD and 44 (64.7%) of eyes reached endpoint for RNFL thickness (as shown by one pair of points in Figure 3); in total 58 EG eyes reached an endpoint by this criterion for either MPD or RNFL thickness (or both). Of these eyes, a larger proportion reached endpoint first by MPD (N=44, 76%) than did by RNFL thickness (N=14, 24%; χ^2^=14.5, two-tailed p=0.0001, McNemar’s test). The odds ratio was thus 3.1, with a 95% confidence interval ranging from 1.7 to 6.2 times more likely to reach endpoint first by MPD than by RNFL thickness. For this criterion, the median time to reach MPD endpoint (N=52) was 152 days post laser, whereas the median time to reach RNFL thickness endpoint (N=44) was 186 days post laser (p=0.03, Mann Whitney test). Considering only those eyes in which *both* MPD and RNFL thickness reached endpoint (N=38), the median time to reach endpoint was 134 days post laser for MPD and 186 days post laser for RNFL thickness (p=0.005, Wilcoxon test). Applying survival analysis whereby eyes without any endpoint were censured at sacrifice revealed that median survival was 191 days for MPD and 274 days for RNFL thickness (χ^2^=10.0, p=0.0015, Log-rank Mantel-Cox Test and χ^2^=13.4, p=0.0003, Gehan-Breslow-Wilcoxon Test). 

**Figure 4:**
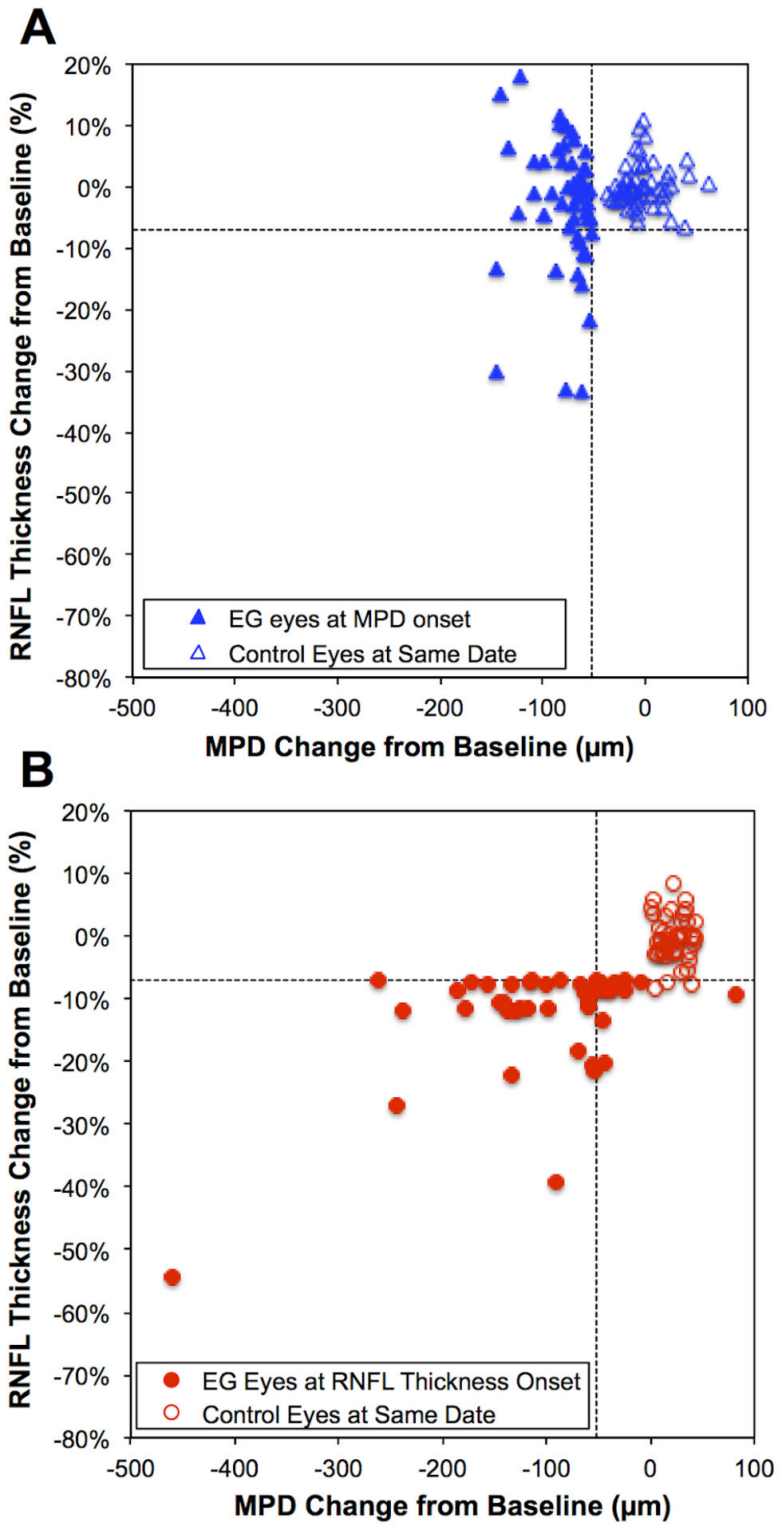
Comparison of RNFL thickness and MPD at onset of change for each parameter. (**A**) The magnitude of change in RNFL thickness from baseline is plotted against the change in MPD from baseline for the 52 EG eyes that reached MPD endpoint (filled triangles) and their fellow control eyes (open triangles). By definition, all of the EG eyes have an MPD change greater than 52 µm (vertical dashed line). At this first, specific, detectable MPD change, the median RNFL thickness change was only 0.9% below from baseline and only 13 (25%) of EG eyes exhibited an RNFL thickness change beyond the measurement noise limit of -7% (horizontal dashed line). (**B**) The same parameters as in panel A are plotted for the 44 EG eyes that reached RNFL thickness endpoint and their fellow control eyes at the onset of RNFL thickness change (the session immediately prior to RNFL thickness endpoint). By definition, all of the EG eyes have an RNFL thickness decline greater than 7% (horizontal dashed line); in this case, three of the control eyes exhibited change this large or greater. At this first, specific, detectable change in RNFL thickness, MPD had already moved posteriorly from baseline by an average of 101 µm and 32 (71%) of EG eyes exhibited a posterior MPD shift larger than the measurement noise limit of 52 µm (vertical dashed line).

Figure 4A shows the change in RNFL thickness from baseline for the 52 EG eyes that reached an MPD endpoint (filled triangles) and their fellow control eyes (open triangles) plotted against the change in MPD from baseline at onset (i.e., the first of the two sequential observations below the measurment noise limit, the second of which defined the endpoint). By definition, at onset, all of the EG eyes have an MPD change greater than 52 µm and for this criterion, none of the control eyes exhibited a change this large. Note, however, at this first, specific detectable MPD change, that RNFL thickness had changed by only -2.2%, on average, from baseline (median: -0.9%, not significantly different from zero, p=0.29, Wilcoxon signed rank test) and that only 13 (25%) of EG eyes exhibited an RNFL thickness loss beyond the measurement noise limit of -7%. 

Figure 4B shows the change in RNFL thickness from baseline for the 44 EG eyes that reached an RNFL thickness endpoint (filled circles) and their fellow control eyes (open circles) plotted against the change in MPD from baseline at onset (i.e., the first of the two sequential observations defining endpoint). Again, by definition, at onset, all of the EG eyes must have reached an RNFL thickness decline greater than 7%; however, in the case of RNFL thickness, three of the control eyes also exhibited change this large or greater at this particular time point. Thus, at this first, specific detectable change in RNFL thickness, MPD had already moved posteriorly from baseline by an average of 101.3 µm (median: 79 µm posterior, p<0.0001, significantly different from zero) and 32 (71%) of EG eyes exhibited a posterior MPD shift larger the measurement noise limit of 52 µm. 

There was no age difference between the group of 14 animals that reached RNFL thickness endpoint first (average age ± SD: 9.0 ± 4.1 years) and the group of 44 animals that hit MPD endpoint first (average age ± SD: 10.2 ± 5.8 years; p=0.55, Mann Whitney test). The difference between endpoint durations for RNFLT and MPD (N=38) was not associated with age (R^2^ = 0.005, p=0.69). 

## Discussion

The results of this study show that the majority of non-human primate eyes with experimental glaucoma demonstrate ONH deformation measurable as a posterior shift in its mean surface height, prior to any detectable change in average peripapillary RNFL thickness measured by SDOCT. The magnitude of the ONH surface height changes are also much larger than can be explained exclusively on the basis of losing axons from both the RNFL and the prelaminar ONH, suggesting that other aspects of ONH deformation such as remodelling and architectural changes of the connective tissue structures within and around the ONH are likely preceding degeneration of axons (at least as can be measured within the adjacent peripapillary retina). For example, since axons bend to some degree as they enter the ONH, the thickness of each individual axon will contribute more to the average surface height of the disc than it does to the thickness of the peripapillary RNFL. This allows the possibility that the change in MPD could be explained simply by the geometry of this bend and loss of axons from both the RNFL and the prelaminar ONH simultaneously. To address this question, we calculated the proportion of mean MPD to mean RNFL thickness changes at the time points shown in Figure 4 and found that the only way in which axon loss alone could explain the MPD change is if the bend at the disc margin was immediate and extremely acute (≥86 deg). Indeed, even if this analysis is limited to those 14 EG eyes that reached endpoint by RNFL thickness prior to MPD, it shows that RNFL thickness was reduced by an average of 13.6 µm at the RNFL thickness endpoint, whereas MPD had changed by an average of -50.2 µm, a proportion that could be explained by the axon loss hypothesis only if the axons abruptly turned ≥75 deg immediately upon crossing the disc margin. Thus, even in the eyes in which the RNFL thickness endpoint occurred before the MPD endpoint, the discrepancy in the absolute magnitude of each change suggests that aspects of ONH deformation beyond simply loss of axons contribute to the early change in ONH surface topography measured here using the parameter MPD. It is also possible that isolated thinning of axon bundles within the prelaminar ONH is contributing to the apparent discrepancy, which could include axon thinning due to chronic “stretch” and/or axon loss that is more advanced within the ONH than in the peripapillary retina (e.g. if axon “die-back” is occurring from the site of the injury at the level of the laminar cribrosa back toward retinal ganglion cell soma). In support of the latter possibility, we have recently reported that axon loss measured in the orbital optic nerve was 10-15% worse than that estimated from SDOCT measurements of RNFL thickness in a subset of these same animals [41]. 

Results of the current quantitative analysis are consistent with the hypothesis that ONH structural deformation precedes axon loss in this experimental model in non-human primates. The results suggest further that ONH structural deformation is an “upstream” event in the pathophysiology of glaucoma, at least in this model. The hypothesis that axons are injured within the ONH by glaucomatous deformation has been proposed and evaluated before [1,2,5,42,43], and extended more recently to rodent models [44,45] (c.f. Chauhan et al. [46]), so the results of this study can be considered confirmatory and to have extended the evidence provided by earlier studies. Interestingly, one parallel hypothesis held that reduced blood flow may be contributory to glaucomatous optic nerve damage, but we have recently reported that ONH blood flow is actually elevated above baseline (and that of fellow control eyes) at an early stage in this experimental glaucoma model, prior to loss of 10% or more of RNFL thickness [47].

One of the limitations of this study is that it included only “global” measurements (i.e. mean position of the entire ONH surface and the average of the entire peripapillary RNFL thickness). It is possible that analysis with finer spatial resolution, such as for quadrants or sectors, might reveal a different result. For example, it was observed in a small number of eyes that focal areas of ONH deformation appreciable by HRT TCA (what might be seen clinically as a focal “notch” in the ONH rim tissue) were associated with relatively limited RNFL bundle defects whereby the global average TSNIT value changed relatively more than did the MPD parameter. However, in the vast majority of EG eyes in this model, ONH and RNFL changes tend to be more diffuse than focal. Nevertheless, for this reason and others, it is imporant to be cautious when generalizing experimental results to clinical glaucoma in human patients. 

In this study, most but not all EG eyes progressed first by MPD, but about one fourth progressed first by RNFL thickness. As mentioned above, in this group of 14 EG eyes, the average MPD change at the RNFL thickness endpoint was -50.2 µm (range -3.3 to -145.3 µm, all posterior to average baseline values), suggesting that the MPD change was close to being signficant (-52 µm) even in about half of these eyes. Nevertheless, these eyes are interesting insofar as they might represent evidence contrasting the hypothesis that ONH deformation precedes axon loss. However, it is possible that in these eyes other aspects of ONH deformation were already present but offsetting or masking posterior change of the average ONH surface topography [26,27,48-50]. One theory posits that older eyes will exhibit relatively less fixed connective tissue deformation and that “cupping” in those eyes would be influenced relatively more by axon loss [25]. We evaluated the influence of age in this study, but found that it did not alter the pattern of findings: older age was not associated with any decreasing gap between MPD and RNFL thickness endpoint nor was age any different in the group of animals whose EG eyes progressed first by RNFL thickness as compared with those progressing first by MPD. This negative result could be due in part to the fact the age range was narrow in this study except for four very young (~2 years) and four much older animals (>20 years), which might have been too small to detect signficant age effects. Separate studies specifically designed to address the influence of age are ongoing. 

In summary, this study demonstrates that in the majority of non-human primate eyes with experimental glaucoma, the average surface height of the ONH measured as the parameter MPD changed prior to any detectable loss of average peripapillary RNFL thickness measured by SDOCT. The magnitude of the ONH surface height changes were also much larger than can be explained exclusively on the basis of losing axons from both the RNFL and the prelaminar ONH. Taken together, these results indicate that ONH deformation manifesting as posterior changes in surface position occur prior to axon loss from the peripapillary RNFL, providing evidence that such deformations are “upstream” pathophysiological events. 
